# Microcephaly Caused by Lymphocytic Choriomeningitis Virus

**DOI:** 10.3201/eid2309.170775

**Published:** 2017-09

**Authors:** Maia Delaine, Anne-Sophie Weingertner, Antoine Nougairede, Quentin Lepiller, Samira Fafi-Kremer, Romain Favre, Rémi Charrel

**Affiliations:** Hôpitaux Universitaires de Strasbourg, Strasbourg, France (M. Delaine, A.-S. Weingertner, Q. Lepiller, S. Fafi-Kremer, R. Favre);; Aix-Marseille University, Marseille, France (A. Nougairede, R. Charrel);; Public Hospitals of Marseille, Marseille (A. Nougairede, R. Charrel)

**Keywords:** lymphocytic choriomeningitis virus, LCMV, viruses, arenavirus, Zika virus, microcephaly, pregnancy, prenatal diagnosis, fetus, infection, PCR, hydrocephalus, meningitis/encephalitis, ultrasonography, ascites, France

## Abstract

We report congenital microencephaly caused by infection with lymphocytic choriomeningitis virus in the fetus of a 29-year-old pregnant women at 23 weeks’ gestation. The diagnosis was made by ultrasonography and negative results for other agents and confirmed by a positive PCR result for lymphocytic choriomeningitis virus in an amniotic fluid sample.

Lymphocytic choriomeningitis virus (LCMV) is an arenavirus, discovered by Armstrong and Lillie in 1933 (*1*) that chronically infects small rodents. Humans can be infected by direct contact with rodents or their fomites, or by inhaling aerosolized particles (*2*). In immunocompetent adults, LCMV infection leads to an influenza-like illness or aseptic meningitis that usually resolves spontaneously; infection can also be asymptomatic (*3*).

When women are infected during pregnancy, the virus can be transmitted to the embryo or fetus transplacentally. Infection causes risk for miscarriage; in utero fetal death; fetopathy, including severe central nervous system or ocular malformations; and severe neurologic sequelae.

Little is known about the incidence and prevalence of LCMV. The association of Zika virus and microcephaly has been reviewed (*4*). Therefore, it is essential to emphasize that other viruses acquired during pregnancy can cause microcephaly and must be considered in differential diagnoses. We report a case of microcephaly caused by LCMV that was diagnosed prenatally.

## The Study

The patient was a 29-year-old G1P0 pregnant woman hospitalized at 23 weeks’ gestation after routine ultrasonography because of fetal ascites and minor ventriculomegaly. Her medical history was unremarkable. The woman and her husband were farmers. Written informed consent was obtained from the patient for participation in this study.

Ultrasonography of the fetus showed symmetric ventriculomegaly and hyperechogenicity of the cerebral parenchyma (Figure). It also detected ascites, a minor pericardial effusion, and cardiomegaly with a hyperechogenic myocardium. The medium cerebral artery peak systolic velocity was 1.98 multiples of median, which indicated fetal anemia. Subsequent ultrasonography showed a rapid increase in ventriculomegaly, cortical atrophy, growth of ascites, and episodes of bradyarrhythmia.

**Figure:**
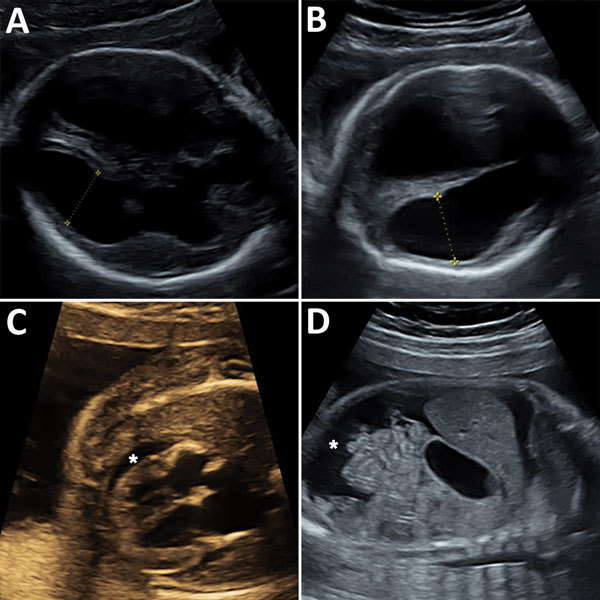
Ultrasonography of congenital microencephaly caused by infection with lymphocytic choriomeningitis virus diagnosed in the fetus of a 29-year-old pregnant women at 23 weeks’ gestation. A) Fetal brain at 23 weeks’ gestation showing symetric ventriculomegaly (14 mm). Yellow symbols indicate axis at which size of cerebral ventricle was measured. B) Fetal brain at 26 weeks’ gestation showing symetric ventriculomegaly (20 mm) and thinning of the cortical mantle. Yellow symbols indicate axis at which size of cerebral ventricle was measured. C) Fetal heart at 24 weeks’ gestation showing pericardial effusion (*) and cardiomyopathy with hyperechogenic muscle. D) Sagittal section of fetal abdomen at 26 weeks’ gestation showing ascites (*).

An initial diagnosis of congenital infection with parvovirus B19 was rejected because maternal serologic results were negative for this virus. Serologic test results were also negative for cytomegalovirus, rubella virus, *Toxoplasma gondii*, and *Treponema pallidum*. The patient had been vaccinated against varicella virus.

We performed amniocentesis at 24 weeks’ gestation: results showed a standard karyotype (46, XX). Results of PCR screening of amniotic fluid were negative for TORCH agents (**t**oxoplasmosis/*Toxoplasma gondii*, **o**ther infections, **r**ubella virus, **c**ytomegalovirus, **h**erpes simplex virus-2 or neonatal herpes simplex virus), as well as enterovirus, *Listeria monocytogenes*, *Mycoplasma* spp., and *Ureaplasma* spp. The biochemical profile of ascites indicated an infection. A fetal blood sample showed moderate anemia. Because of these negative results for virus infections, an ascitic fluid sample was tested by PCR for LCMV.

We extracted virus RNA from an ascite sample by using a Z1-XL Biorobot and a Virus Mini Kit (QIAGEN, Hilden, Germany). A 253-nt region in the small RNA segment was amplified by using sense primer CML-F0 (5′-ARCAARGGIATYTGTAGYTGTGG-3′) and reverse primer CML-R3 (5′-CTYATGGAYTGCATCATYTTTGA-3′) in a QuantiTect SYBR Green Real-Time PCR Device (QIAGEN), a CFX96 thermal cycler (Bio-Rad Laboratories, Hercules, CA, USA), and the cycling protocol reported for flaviviruses (*5*).

To better characterize this strain, a 686-bp product was amplified by using primers for the virus polymerase gene (*6*) and directly sequenced. We used this sequence for alignment with homologous virus sequences in GenBank and performed phylogenetic analysis by using the maximum-likelihood method based on the Kimura 2-parameter model implemented in MEGA 6.06 software (*7*).

We tested 5 samples for virus small gene segment by using a specific Sybr Green Real Time PCR. Amniotic fluid was positive for LCMV. Fetal brain and placenta biopsy specimens and a serum sample from the mother were negative for LCMV. The matching sequence was compared with 42 other LCMV sequences by using BLAST (https://blast.ncbi.nlm.nih.gov/Blast.cgi). Genetic identities ranged from 77.2% to 90.8% at the nucleotide level.

For confirmation and better genetic characterization, we tested samples by using a reverse transcription PCR specific for virus large gene segment. Again, only amniotic fluid was positive. The 686-nt sequence matched 31 other sequences in GenBank (identity range 78.2%–88%). All specimens were inoculated onto Vero cells, but virus was not isolated.

The patient reported an influenza-like illness during the 16th week of pregnancy, which had resolved spontaneously in a few days. She also reported that there were mice on the farm.

On the basis of echographic findings, the couple decided to terminate the pregnancy at 28 weeks’ gestation. A fetal blood sample showed increased anemia and thrombocythemia.

Fetopathologic examination showed hepatosplenomegaly, thymic hypertrophy, ascites, and pericardial and pleural effusion. Examination of the brain showed severe microcephaly with polymicrogyria, a thin cortex, and diffuse periventricular calcifications. We also detected bilateral chorioretinitis. The placenta was unremarkable, and results of genetic analysis were within reference ranges.

## Conclusions

The prevalence of LCMV appears to be low (LCMV IgG 0.3%) in France (*8*). Only 4 cases of LCMV infection have been described in France since 1978 (*9**–**11*). The laboratory of clinical microbiology at Public Hospitals of Marseille has received 83 samples for detection of LCMV during 2015–2016. The only other amniotic fluid specimen tested during this period was negative for LCMV by PCR. During 2005–2015, this laboratory has not reported a positive result for LCMV (R. Charrel, Aix-Marseille University, Marseille, France, 2017, pers. comm).

It is difficult to determine whether these findings were caused by low circulation of LCMV or lack of awareness of general practitioners and obstetricians. However, although Zika virus has been recently identified as a cause of microcephaly, physicians should consider other viruses when dealing with signs compatible with congenital infection by Zika virus.

Since 1955, a total of 58 cases of congenital LCMV infections have been reported worldwide; all were diagnosed postnatally (*2**,**10**,**12**,**13*). An influenza-like illness was described in 50% of pregnant women, and exposure to rodents was reported by 33% (*2**,**10**,**12**,**13*). Chorioretinitis and chorioretinal scars were observed in 89%–100% of infected children, and hydrocephalus (mostly triventricular dilation) in 96% (*2**,**10**,**12**,**13*).

There are many prenatal ultrasonic signs of LCMV infection, involving mostly the central nervous system. Of these signs, ventriculomegaly is the most common. Bilateral cataracts are also observed. The estimated mortality rate for infants with prenatal LCMV infection is 30%–35% at the age of 21 months (*14**,**15*). Almost all survivors have neurologic sequelae (*14*), of which 67% are severe (*2**,**13**,**15*).

Differential diagnoses of congenital LCMV infection include testing for TORCH infections (Table). A definitive diagnosis relies on virus identification by serologic analysis or direct evidence, such as virus isolation or detection of LCMV RNA in fetal or maternal samples. For our patient, limited volumes of samples precluded additional serologic analysis; thus we performed testing by PCR. However, a positive PCR result and sequence confirmation are direct evidence for the presence of LCMV.

**Table:** Characteristics of neonates with congenital viral or bacterial infections, including a fetus with congenital microencephaly caused by infection with LCMV in a 29-year-old pregnant women at 23 weeks’ gestation*

Virus infection or disease	VM	Intracranial hypertension	Calcification	Microcephaly	Retinopathy	Hearing impairment	HSM	Nonimmune anasarca	Fetal growth restriction
LCMV	+++	+	++++	+++	+++	?	?†	+	?‡
Toxoplasmosis	+	+	++	+	+++	+	++	+	+
Rubella virus	–	+	±	+	+	+++	+++	±	+
CMV	–	+	+	+++	+	+++	+++	+	+
HSV	–	+	+	+	+	–	+	+	+
Syphilis	±	+	–	–	+	+	+++	+	+
Parvovirus B19	–	–	–	–	–	–	+	++	+
Zika virus§	++	+	+	+++	+	?	?	+	++

*Data were obtained from Anderson et al. (*2*) and Barton and Mets (*3*). CMV, cytomegalovirus; HSM, hepatomegaly/splenomegaly; HSV, herpes simplex virus; LCMV, lymphocytic choriomeningitis virus; VM, ventriculomegaly. –, not observed; ±, possibly observed; +, rarely observed; ++, sometimes observed; +++, frequently observed; ++++, constantly observed; ?, not known. †Diagnosed only by postmortem examination (*8*; this study). ‡Reported by Bonthius (*14*) in 6 of 20 neonates.  §Reported by Alvarado and Schwartz (*4*).

When ultrasonographic signs suggestive of infection are identified, complete ultrasonography can be performed to identify associated abnormalities and conventional congenital infections. If results of this initial assessment are negative, testing for LCMV is indicated for fetal samples and maternal serum samples. Medical termination of the pregnancy might need to be considered for some cases.

No vaccine or effective treatment is available for infection with LCMV. Ribavirin has been used for some cases of severe infection. However, this drug is contraindicated for pregnant women (*12*). For these women, only preventive measures are available.

LCMV infection is not included among occupation-related diseases in France, and there are few case reports of infection with this virus. Large-scale prospective studies are needed to determine the incidence of malformations associated with this virus. Lesions caused by LCMV might cover a broad spectrum, ranging from minor to severe and irreversible manifestations. Thus, LCMV infection should be considered a possible etiology requiring laboratory investigations for cases of evocative neurologic malformations or nonimmunologic anasarca not caused by a TORCH infection or genetic or chromosomal abnormalities.
